# Refining orthologue groups at the transcript level

**DOI:** 10.1186/1471-2164-11-S4-S11

**Published:** 2010-12-02

**Authors:** Yizhen Jia, Thomas KF Wong, You-Qiang Song, Siu-Ming Yiu, David K Smith

**Affiliations:** 1 Department of Biochemistry, The University of Hong Kong, Hong Kong; 2 Department of Computer Science, The University of Hong Kong, Hong Kong

## Abstract

**Background:**

Orthologues are genes in different species that are related through divergent evolution from a common ancestor and are expected to have similar functions. Many databases have been created to describe orthologous genes based on existing sequence data. However, alternative splicing (in eukaryotes) is usually disregarded in the determination of orthologue groups and the functional consequences of alternative splicing have not been considered. Most multi-exon genes can encode multiple protein isoforms which often have different functions and can be disease-related. Extending the definition of orthologue groups to take account of alternate splicing and the functional differences it causes requires further examination.

**Results:**

A subset of the orthologous gene groups between human and mouse was selected from the InParanoid database for this study. Each orthologue group was divided into sub-clusters, at the transcript level, using a method based on the sequence similarity of the isoforms. Transcript based sub-clusters were verified by functional signatures of the cluster members in the InterPro database. Functional similarity was higher within than between transcript-based sub-clusters of a defined orthologous group. In certain cases, cancer-related isoforms of a gene could be distinguished from other isoforms of the gene. Predictions of intrinsic disorder in protein regions were also correlated with the isoform sub-clusters within an orthologue group.

**Conclusions:**

Sub-clustering of orthologue groups at the transcript level is an important step to more accurately define functionally equivalent orthologue groups. This work appears to be the first effort to refine orthologous groupings of genes based on the consequences of alternative splicing on function. Further investigation and refinement of the methodology to classify and verify isoform sub-clusters is needed, particularly to extend the technique to more distantly related species.

## Background

Orthologous genes are related to each other through divergent evolution from a common ancestor and so are expected to have similar functions. Determining orthologous gene groups, where at least one member has a known function, is a common method to extend functional annotation to genes in other species [1,2]. Existing approaches to identify orthologues are mainly based at the protein level, using a representative or the longest transcript of a gene [2]. However, recent studies suggest that more than 90% of human multi-exon protein coding-genes are involved in the process of alternative splicing [3,4]. As alternative pre-mRNA splicing is considered a key mechanism to generate structural and functional complexity in higher eukaryotes, alternative transcripts of a gene may have differing functional roles [5]. Consequently, the assignment of function across an orthologous gene group might not apply to alternate transcripts of the genes.

It has been shown that different protein isoforms generated by alternative splicing can have diverse functional properties, such as binding characteristics, subcellular localisation or enzymatic activity [6] or have altered structural properties [6] or tissue specificity [3]. In many cases, the isoforms of proteins within an orthologous grouping might not meet the basic assumption of functional similarity for all combinations of isoforms. Indeed inappropriate expression of an alternate isoform of a gene can be a significant cause of disease [7,8].

Intronic mutations and synonymous SNPs can affect splicing resulting in disease by inappropriate expression of an isoform [7-9]. For example, tauopathies can be caused by an exon 10 mutation altering the relative expression levels of two isoforms (3R and 4R) of the MAPT gene [7,8]. Differing isoforms of VEGF-A have either pro- or anti-angiogenic properties and so provide a possible target for cancer therapy [10,11]. In some cancerous tissues isoforms of a gene that are not found in normal tissues are expressed [12]. A clearer understanding of the functional relationships among isoforms in orthologous gene sets will be helpful to investigating pathological conditions.

Several databases and resources have been created to generate orthologous gene groups over a large range of species (e.g. [1,2,13,14]) and comparative studies of orthologue databases have been made [15]. These resources focus on protein sequence alignments without fully taking account of alternatively spliced isoforms. EnsemblCompara [2], for example, selects the longest transcript of a gene. Recently the “GOOD” database [16,17] was constructed to define orthologue groups by examining the “processed transcription units” of a gene (the genomic region that encompasses all exons of a gene whether alternate or constitutive). This method provided better coverage of orthologue groups and distinction between orthologues and paralogues [16,17].

This work seeks to refine, not define, orthologous groupings of genes by arranging them into subsets where the differing functional properties of alternate transcripts are accounted for. Currently available definitions of orthologue groups are taken and those that have multiple isoforms are sub-clustered by a simple technique into groups with related sequence similarity. These sub-clusters are then subjected to verification tests based on functional and structural considerations to show that the clustering technique does provide biologically meaningful subdivisions of existing orthologue groups.

Similar to Ho et al [16,17], and unlike earlier orthologue finding procedures, this work explicitly focuses on the alternate splicing in eukaryotes. However, this work is not concerned with the definition of orthologue clusters at the overall gene level, but with the refinement of orthologue clusters to account for the expansion of function that alternative splicing allows. This will provide biologists with a family of refined orthologue groupings which will allow more precise experimentation into the particular functions of an isoform and its “orthologous isoforms”.

## Methods

### Data sources

Human and mouse orthologue data were downloaded from InParanoid 7.0 (June, 2009) [1]. Only gene groups that showed a one-to-one orthologue relationship between human and mouse were retained. Protein sequences for these genes were obtained from Ensembl release 56 (September, 2009) giving a total of 11,854 orthologous gene groups that contained at least one human and one mouse protein. Functional descriptions were taken from the InterPro database [18] using the InterProScan tool [19]. Data on disordered regions in proteins, in part, used the DisProt database (release 4.9, June 2009) [20]. However there are only limited data in this database of experimentally verified results and only 193 human proteins had annotations available.

### Clustering method

Protein products of all the alternately spliced isoforms of an orthologous gene pair were multiply aligned using the program MUSCLE [21]. Once aligned, all the common regions among these proteins could be identified. For any two proteins *P1* and *P2*, the similarity score between them was defined as: Sim (P1,P2) = (number of identities or substitutions / total aligned length including gaps). Therefore, 0 ≤ Sim (P1,P2) ≤ 1. Due to the heuristic nature of multiple sequence alignment programs, some regions may not have aligned correctly. To adjust for this, common regions that have length < ‘d’ were removed from consideration. Currently, ‘d’ was set at 2.

After computing all the pair-wise similarity scores between protein isoforms, sub-clusters were built similarly to the method used in InParanoid [22]. The best matched isoforms across two species were marked as the anchor of an orthologous transcript sub-cluster. Then additional isoforms were added to the sub-cluster if the similarity score between an isoform and its anchor in the same species is higher than the similarity score of the two anchor proteins. The process repeats for the remaining sequences. Some isoforms may form singleton sub-clusters.

**Figure 1:**
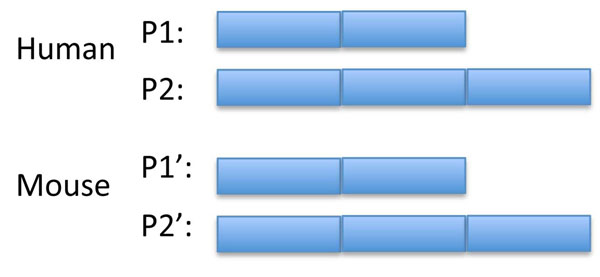
**Illustration of the alternative transcript similarity scoring scheme.** For an orthologue group with two alternative transcripts in each species, a global multiple sequence alignment results in the alignment of homologous exons. Where an alignment of isoforms that differ in the number of exons is used to calculate the similarity score (eg P1-P2') a score <1 will be obtained (0.67 in this case if the exons are assumed to have equal length) as the extra exon is matched by a gap in the alignment. P1-P1' and P2-P2' will give scores of 1.

An example calculation is shown in Fig. 1 for protein products of orthologous genes in species A and B where each gene has two isoforms which differ by the presence of a third exon in one isoform. If, for simplicity, the exons are assumed to have the same length, then

Sim (P1, P1') = Sim (P2, P2') = 1,

Sim (P1, P2') = Sim (P2, P1') = 2/3, and

Sim (P1, P2) = Sim (P1', P2') = 2/3.

So P1 and P1' will form a transcript sub-cluster, which will not be expanded as the in-species isoform scores are less than the anchor pair’s score. Likewise P2 and P2' will form a transcript sub-cluster. Thus the original single gene orthologue pair has become two transcript-based orthologue sub-clusters. It should be noted that a local alignment based scoring technique such as BLAST would not distinguish between the P1-P1' and P1-P2' pairings.

### Functional and disorder difference measures

Two functional and structural methods to assess the efficacy of the orthologue sub-clustering method were utilised. One was domain based signatures from the InterPro database [18] and the second was the presence of (predicted) regions of intrinsic disorder in the proteins as protein disorder has functional associations with splicing [23].

InterProScan [19] was used to identify all the signatures that matched to any of the transcripts in an orthologous gene cluster. For each transcript a vector was constructed indicating whether that transcript matched (1) or did not match (0) that signature. Then the functional difference difff_func_() of two transcripts was defined to be the Hamming distance between their two signature vectors (or the count of the number of functional signatures at which they differed).

Due to the limited amount of experimental data on protein disorder in the DisProt [20] database, predictions of disorder were made for each transcript using PONDR VSL2B [24,25]. The default values were used to define potential disordered regions and the number and length of disordered regions were kept for each isoform in an orthologous gene cluster. Short predictions of disorder (< 10 amino acids) were ignored and disordered regions separated by < 3 amino acids were combined. For two isoforms, diff_dis_() was defined as the absolute value of the difference in the number of disordered regions between them.

Average values of the difference measures were calculated within (intra) and between (inter) sub-clusters of an orthologue group and compared. Wilcoxon ranked sum tests were used to assess the distribution of average values between the intra- and inter-sub-group differences.

## Results

### Sub-clustering

11,854 orthologous one-to-one gene groups, for which at least one protein sequence from each species could be obtained from Ensembl, were taken from InParanoid. The numbers of protein isoforms per group are shown in Table 1. 6,085 orthologous groups contained only 2 sequences and so could not be sub-clustered. The remaining 5,769 groups were further processed by applying the clustering approach outlined in the Methods section. 3,421 orthologous gene groups (28.9% of total or 59.3% of clusters with ≥ 3 proteins) could be divided into 2 or more sub-clusters (Table 2).

**Table 1:** The distribution of numbers of proteins in an orthologue group

Protein Group Size	Number
2	6,085
3	2,703
4	1,413
5	748
6	347
7	230
>8	328

Total	11,854

**Table 2:** Numbers of sub-clusters by orthologue group

Number of sub-clusters	Number of gene orthologue groups		Total

	3 proteins in group	> 3 proteins in group	
Only 1 cluster	1,391 (51.5%)	887 (28.9%)	2,278 (39.5%)

≥ 2 sub-clusters	1,312 (48.5%)	2,109 (71.1%)	3,491 (60.5%)

### Functional annotations

**Figure 2:**
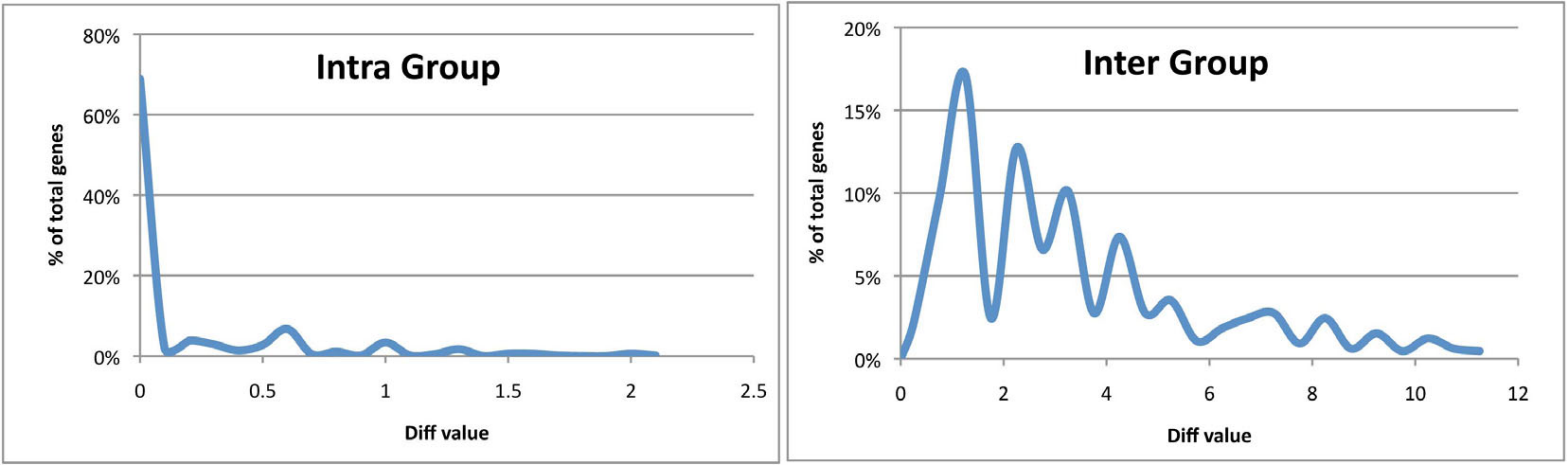
The distribution of the average diff_func_() values for each orthologous gene set for intra-group (left) and inter-group (right) comparisons.

Orthologue groups that were sub-clustered were verified for functional consistency by using InterProScan to identify signatures in the sequences. No differences were found in this functional signature among the sub-clusters for 65.7% of the orthologue groups, perhaps reflecting the limits of current annotation processes for alternately spliced isoforms. Where differences were found, the intra- and inter-group difference scores were calculated for all the sub-clusters of the orthologue groups. These diff_func_() values are given in Fig. 2 for the intra- and inter-groups. In approximately 70% of the cases the intra-group difference was 0. Mean scores for intra- and inter-group values of diff_func_() were 0.27 and 4.13, respectively. A Wilcoxon ranked sum test of the difference in the distributions of diff_func_() scores showed a statistically significant difference between the goups with a p-value of ≤ 2.2×10^-16^.

### Intrinsic disorder predictions

**Figure 3:**
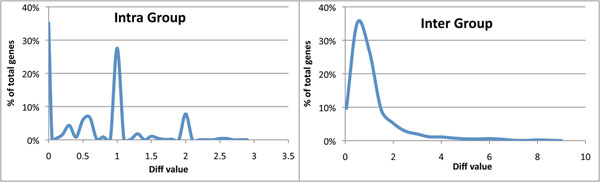
The distribution of the average diff_dis_() values for each orthologous gene set for intra-group (left) and inter-group (right) comparisons.

After predicting the intrinsically disordered regions in all the protein isoforms of the sub-clustered orthologue groups, it was found that 25% of the groups had identical disorder predictions. As for the functional annotations, all diff_dis_() values were calculated for intra- and inter-group comparisons in the remaining orthologue group sub-clusters. Fig. 3 shows the distribution of these values. About 35% of the intra-group comparisons showed no difference in diff_dis_() values. Average scores for the intra- and inter-group diff_dis_() values were 0.76 and 1.37 respectively. The distributions of intra- and inter-group values were found to be statistically significantly different by a Wilcoxon ranked sum test with a p-value of ≤ 2.2×10^-16^.

### Specific examples of the method

**Figure 4:**
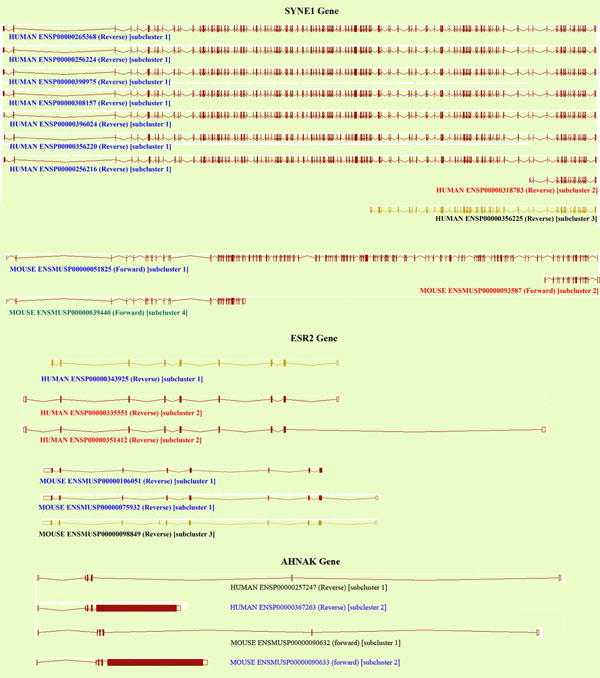
The gene structures of SYNE1, ESR2 and AHNAK.

Application of the techniques described here is shown for the genes SYNE1, ESR2 (ERβ), and AHNAK. Figure 4 shows the exon/intron structure for the isoforms of these genes which formed 4, 3 and 2 sub-clusters for SYNE1, ESR2 and AHNAK respectively. Table 3 shows the InterPro functional annotation patterns for these genes in their sub-clusters while Table 4 gives the details of disordered regions by gene and sub-cluster. In SYNE1 the sub-clusters show different functional and disorder patterns from each other except for the InterPro functions of sub-clusters 2 and 3 which are identical. ESR2 forms 3 sub-clusters which separate the normal tissue form from the cancer associated form [26] and all 3 sub-clusters show differences in the degree of predicted intrinsic disorder. AHNAK shows 2 clusters with marked differences in the amount of disorder.

**Table 3:** InterPro keyword labels of the protein isoforms of genes SYNE1, ESR2 and AHNAK

Gene	Cluster#	Protein	SYNE1's keyword labels *																
SYNE1	1	ENSMUSP00000051825	1	1	1	1	1	1	1	1	1	1	1	1	1	1	1	1	1
	1	ENSP00000265368	1	1	1	1	1	1	1	1	1	1	1	1	1	1	1	1	1
	1	ENSP00000308157	1	1	1	1	1	1	1	1	1	1	1	1	1	1	1	1	1
	1	ENSP00000356216	1	1	1	1	1	1	1	1	1	1	1	1	1	1	1	1	1
	1	ENSP00000356220	1	1	1	1	1	1	1	1	1	1	1	1	1	1	1	1	1
	1	ENSP00000356224	1	1	1	1	1	1	1	1	1	1	1	1	1	1	1	1	1
	1	ENSP00000390975	1	1	1	1	1	1	1	1	1	1	1	1	1	1	1	1	1
	1	ENSP00000396024	1	1	1	1	1	1	1	1	1	1	1	1	1	1	1	1	1

	2	ENSMUSP00000093587	1	1	0	0	1	1	0	1	0	1	1	0	0	0	1	0	1
	2	ENSP00000318783	1	1	0	0	1	1	0	1	0	1	1	0	0	0	1	0	1

	3	ENSP00000356225	1	1	0	0	1	1	0	1	0	1	1	0	0	0	1	0	1

	4	ENSMUSP00000039440	1	0	1	1	0	0	1	0	1	0	0	1	1	1	0	1	0

			ESR2's keyword labels*																

ESR2	1	ENSP00000343925 (ERβw)	1	1	1	1	1	1	1	1	1	1	1	1	1	1	1	1
	1	ENSMUSP00000075932	1	1	1	1	1	1	1	1	1	1	1	1	1	1	1	1
	1	ENSMUSP00000106051	1	1	1	1	1	1	1	1	1	1	1	1	1	1	1	1

	2	ENSP00000351412 (ERβcx)	1	1	1	1	1	1	1	1	1	1	1	1	1	1	1	1
	2	ENSP00000335551 (ERβcx)	1	1	1	1	1	1	1	1	1	1	1	1	1	1	1	1

	3	ENSMUSP00000098849	1	1	1	1	1	1	1	1	1	1	1	1	1	1	1	1

			AHNAK's keyword labels *																

AHNAK	1	ENSP00000257247	1	1	1	1	0	1	1	1	0
	1	ENSMUSP00000090632	1	1	1	1	0	1	1	1	0

	2	ENSP00000367263	1	1	1	1	0	1	1	0	1
	2	ENSMUSP00000090633	0	1	1	1	1	1	1	0	1

*SYNE1’s keyword labels: GO3779, GO16021, IPR001589, IPR001715, IPR002017, IPR012315, IPR016146, IPR018159, PF00307, PF00435, PF10541, PS00019, PS00020, PS50021, PS51049, SM00033, SM00150

ESR2’s keyword labels: PR00350, PF00104, SM00430, PR00047, PF00105, SM00399, PS00031, PS51030, PR00398, G3DSA:1.10.565.10, SSF48508, G3DSA:3.30.50.10, PF12497, PTHR11865, PTHR1186:SF216, SSF57716

AHNAK’s keyword labels: PF00595, SM00228, PS50106, SSF50156, PS01031, G3DSA:2.30.42.10, PTHR23348, PRHR23348:SF7, PTHR23348:SF8

**Table 4:** The prediction of disordered regions in the protein isoforms of genes SYNE1, ESR2 and AHNAK

Gene	Cluster#	Protein	# of disordered regions	Sum of region lengths	Protein length	Proportion of disordered regions
SYNE1	1	ENSMUSP00000051825	94	4390	8800	49.9%
	1	ENSP00000265368	89	4326	8798	49.2%
	1	ENSP00000308157	88	4275	8750	48.9%
	1	ENSP00000356216	89	4327	8798	49.2%
	1	ENSP00000356220	88	4276	8750	48.9%
	1	ENSP00000356224	89	4372	8798	49.7%
	1	ENSP00000390975	88	4321	8750	49.4%
	1	ENSP00000396024	88	4275	8750	48.9%

	2	ENSMUSP00000093587	9	410	950	43.2%
	2	ENSP00000318783	9	403	983	41.0%

	3	ENSP00000356225	29	1727	3322	52.0%

	4	ENSMUSP00000039440	16	616	1432	43.0%

ESR2	1	ENSP00000343925	5	243	531	45.8%
	1	ENSMUSP00000075932	5	295	550	53.6%
	1	ENSMUSP00000106051	5	295	550	53.6%

	2	ENSP00000351412	5	244	496	49.2%
	2	ENSP00000335551	5	244	496	49.2%

	3	ENSMUSP00000098849	5	295	568	51.9%

AHNAK	1	ENSP00000257247	1	67	150	44.7%
	1	ENSMUSP00000090632	2	120	185	64.9%

	2	ENSP00000367263	6	5716	5891	97.0%
	2	ENSMUSP00000090633	10	5431	5657	96.0%

## Discussion

With the substantial increase in the number of new genomes being sequenced due to next generation sequencing technology, the need for better functional annotation of genes is becoming more urgent. Automatic assignment of functions based on orthologous gene relationships is one of the key processes in electronic annotation of databases [2]. Much effort has been placed into developing methods to accurately identify orthologous relationships yet, while recent work has considered using transcript data to define gene regions [16,17], alternate splicing has not been considered from the point of view of the different functions isoforms of a gene may have [5].

This work has explicitly considered the alternately spliced isoforms of a gene and the available, if still restricted, functional data on them. The results shown here demonstrate that many orthologous gene groups are comprised of sets of isoforms that have detectable differences in functional attributes. Based on this, automatic assignment of functions from one isoform in a gene based orthologue group to all isoforms in another species could easily result in annotation errors which would likely continue to propagate. If the refinement of the orthologous gene groups defined here is taken into account, then annotations will only be transferred within the isoform sub-clusters of the orthologous gene group to which they apply. This should greatly enhance the information available to clinicians seeking to explain and treat the many pathobiologies involving alternate splicing [7,8].

For this study an existing definition of orthologous groups [1] was taken. Protein isoforms within these groups had to be clustered. Using standard tools like BLAST is not adequate as its local alignment algorithmic base means it will not distinguish between isoforms which differ by the inclusion of additional exons in one isoform (see Fig 1). Consequently, a similarity measured based on global comparisons needed to be used. Although the method used here is computationally simple (the ratio of identities or substitutions to global alignment length) it effectively models the sharing of homologous exons between isoforms as these align with each other. Skipped, mutually exclusive, truncated and other variants of exons will contribute to gaps in one of the sequences in the alignment. Thus the similarity score used here reflects the exon structure of the isoforms.

When InterPro signatures were used to assess the sub-clusters of orthologue groups, many (~65%) had no differences among the sub-clusters. In part this might reflect the nature of InterPro which has a domain based approach to functional annotation. If constitutive exons match the InterPro signatures all isoforms will receive the same annotation even if the presence or absence of other domains might affect the function of the protein. Another aspect of this problem is that most annotation efforts have been focussed at the gene or dominant transcript level and the full extent of alternate splicing is only recently being revealed [3,4]. Different functional annotation methods, or a larger combination of methods, might provide a better separation of the subgroups based on function.

Where the sub-clusters of an orthologue group had differing annotations from InterPro, it was clear that the sub-clusters developed here were reflecting functional differences. The intra-group difference in functional assignment was significantly smaller than the inter-group difference. Most of the intra-group functional difference scores were 0 reflecting the functional consistency of the sub-clusters. Thus the clustering method used here appears to have provided a useful separation of orthologous gene groups into subsets of distinct functionality.

Intrinsic disorder in proteins has been associated with alternate splicing [23] and many aspects of protein function [27]. While experimentally verified definitions of intrinsic disorder are few [20], many tools are available to predict disorder (eg [24,25]). Although, as computational predictions, these suffer from the lack of experimental verification, they avoid the problem of a single gene based approach to function and can be applied to all alternately spliced isoforms. Far fewer (about 13%) of the sub-clusters of an orthologue group showed no difference on this measure. Most of the orthologue groups examined in this work had sub-clusters that showed distinct differences in the extent of intrinsic disorder of their members. As was the case with the InterPro classifications, the intra-group differences in disorder were significantly smaller than the inter-group differences. On this basis the clustering technique developed here has been able to separate orthologue groups into sub-clusters with distinct biological properties.

The InParanoid orthologue groups that could be divided into sub-clusters are available as Additional File 1. From the evidence presented here, researchers who wish to investigate a protein based on functions observed for its orthologue in, for example, a model species should take account of alternate splicing and ensure that they are evaluating orthologues at the transcript level and not just the gene level.

## Conclusions

The work presented here has provided an initial refinement of protein orthologous clusters at the transcript level. While the sub-clustering technique used was relatively simple it has produced sub-clusters of orthologue groups that show distinct biological patterns based on two independent measures. While transcript level data has been used previously to define gene-level orthologue groups, this appears to be the first work to examine and subdivide orthologue gene groups based on their alternatively spliced transcripts which often have differing functions. Based on the results presented here, it seems advisable to extend the concept of orthology from the gene to the transcript level.

Extension of this work to generate orthologous gene sub-clusters across many species will be a critical direction for further investigation. Other transcript level features such as exon usage, tissue specificity, and splicing pattern could be used to improve the reliability of the sub-clustering method. Assigning an increased range of functional parameters to the sub-clusters should improve their utility in guiding experimental work.

## Authors' contributions

TW and SMY conceived the study. YJ, TW and SMY refined the study and YJ and TW implemented it. All authors contributed to the analysis. YJ, TW, YQS and SMY participated in the initial draft. DS wrote the final version. All authors read and approved the final version.

## Competing interests

The authors declare that they have no competing interests

## Supplementary Material

Additional File 1 Transcript based subclusters of human-mouse InParanoid orthologue groupings (in tab-delimited text format). Click here for file

## References

[B1] Berglund AC Sjolund E Ostlund G Sonnhammer EL InParanoid 6: eukaryotic ortholog clusters with inparalogs Nucleic Acids Res 2008 36 Database issue D263 266 18055500 10.1093/nar/gkm1020 PMC2238924

[B2] Vilella AJ Severin J Ureta-Vidal A Heng L Durbin R Birney E EnsemblCompara GeneTrees: Complete, duplication-aware phylogenetic trees in vertebrates Genome Res 2009 19 2 327 335 19029536 10.1101/gr.073585.107 PMC2652215

[B3] Wang ET Sandberg R Luo S Khrebtukova I Zhang L Mayr C Kingsmore SF Schroth GP Burge CB Alternative isoform regulation in human tissue transcriptomes Nature 2008 456 7221 470 476 18978772 10.1038/nature07509 PMC2593745

[B4] Pan Q Shai O Lee LJ Frey BJ Blencowe BJ Deep surveying of alternative splicing complexity in the human transcriptome by high-throughput sequencing Nat Genet 2008 40 12 1413 1415 10.1038/ng.259 18978789

[B5] Nilsen TW Graveley BR Expansion of the eukaryotic proteome by alternative splicing Nature 2010 463 7280 457 463 10.1038/nature08909 20110989 PMC3443858

[B6] Floris M Orsini M Thanaraj TA Splice-mediated Variants of Proteins (SpliVaP) - data and characterization of changes in signatures among protein isoforms due to alternative splicing BMC Genomics 2008 9 453 18831736 10.1186/1471-2164-9-453 PMC2573899

[B7] Tazi J Bakkour N Stamm S Alternative splicing and disease Biochim Biophys Acta 2009 1792 1 14 26 18992329 10.1016/j.bbadis.2008.09.017 PMC5632948

[B8] Ward AJ Cooper TA The pathobiology of splicing J Pathol 2010 220 2 152 163 19918805 10.1002/path.2649 PMC2855871

[B9] Takahashi A Effect of exonic splicing regulation on synonymous codon usage in alternatively spliced exons of Dscam BMC Evol Biol 2009 9 214 19709440 10.1186/1471-2148-9-214 PMC2741454

[B10] Harper SJ Bates DO VEGF-A splicing: the key to anti-angiogenic therapeutics? Nat Rev Cancer 2008 8 11 880 887 18923433 10.1038/nrc2505 PMC2613352

[B11] Rennel ES Harper SJ Bates DO Therapeutic potential of manipulating VEGF splice isoforms in oncology Future Oncol 2009 5 5 703 712 19519209 10.2217/fon.09.33 PMC2879319

[B12] Xu Q Lee C Discovery of novel splice forms and functional analysis of cancer-specific alternative splicing in human expressed sequences Nucleic Acids Res 2003 31 19 5635 5643 14500827 10.1093/nar/gkg786 PMC206480

[B13] Sayers EW Barrett T Benson DA Bolton E Bryant SH Canese K Chetvernin V Church DM Dicuccio M Federhen S Database resources of the National Center for Biotechnology Information Nucleic Acids Res 2010 38 Database issue D5 16 19910364 10.1093/nar/gkp967 PMC2808881

[B14] Li L Stoeckert CJ Jr. Roos DS OrthoMCL: identification of ortholog groups for eukaryotic genomes Genome Res 2003 13 9 2178 2189 12952885 10.1101/gr.1224503 PMC403725

[B15] Altenhoff AM Dessimoz C Phylogenetic and functional assessment of orthologs inference projects and methods PLoS Comput Biol 2009 5 1 e1000262 19148271 10.1371/journal.pcbi.1000262 PMC2612752

[B16] Ho MR Jang WJ Chen CH Ch'ang LY Lin WC Designating eukaryotic orthology via processed transcription units Nucleic Acids Res 2008 36 10 3436 3442 18445630 10.1093/nar/gkn227 PMC2425467

[B17] Ho MR Chen CH Lin WC Gene-oriented ortholog database: a functional comparison platform for orthologous loci Database (Oxford) 2010 2010 baq002 20428317 10.1093/database/baq002 PMC2860896

[B18] Hunter S Apweiler R Attwood TK Bairoch A Bateman A Binns D Bork P Das U Daugherty L Duquenne L InterPro: the integrative protein signature database Nucleic Acids Res 2009 37 Database issue D211 215 18940856 10.1093/nar/gkn785 PMC2686546

[B19] Zdobnov EM Apweiler R InterProScan--an integration platform for the signature-recognition methods in InterPro Bioinformatics 2001 17 9 847 848 10.1093/bioinformatics/17.9.847 11590104

[B20] Sickmeier M Hamilton JA LeGall T Vacic V Cortese MS Tantos A Szabo B Tompa P Chen J Uversky VN DisProt: the Database of Disordered Proteins Nucleic Acids Res 2007 35 Database issue D786 793 17145717 10.1093/nar/gkl893 PMC1751543

[B21] Edgar RC MUSCLE: a multiple sequence alignment method with reduced time and space complexity BMC Bioinformatics 2004 5 113 15318951 10.1186/1471-2105-5-113 PMC517706

[B22] Remm M Storm CE Sonnhammer EL Automatic clustering of orthologs and in-paralogs from pairwise species comparisons J Mol Biol 2001 314 5 1041 1052 10.1006/jmbi.2000.5197 11743721

[B23] Romero PR Zaidi S Fang YY Uversky VN Radivojac P Oldfield CJ Cortese MS Sickmeier M LeGall T Obradovic Z Alternative splicing in concert with protein intrinsic disorder enables increased functional diversity in multicellular organisms Proc Natl Acad Sci USA 2006 103 22 8390 8395 16717195 10.1073/pnas.0507916103 PMC1482503

[B24] Obradovic Z Peng K Vucetic S Radivojac P Dunker AK Exploiting heterogeneous sequence properties improves prediction of protein disorder Proteins 2005 61 Suppl 7 176 182 10.1002/prot.20735 16187360

[B25] Peng K Radivojac P Vucetic S Dunker AK Obradovic Z Length-dependent prediction of protein intrinsic disorder BMC Bioinformatics 2006 7 208 16618368 10.1186/1471-2105-7-208 PMC1479845

[B26] Omoto Y Kobayashi S Inoue S Ogawa S Toyama T Yamashita H Muramatsu M Gustafsson JA Iwase H Evaluation of oestrogen receptor beta wild-type and variant protein expression, and relationship with clinicopathological factors in breast cancers Eur J Cancer 2002 38 3 380 386 10.1016/S0959-8049(01)00383-5 11818203

[B27] Dunker AK Oldfield CJ Meng J Romero P Yang JY Chen JW Vacic V Obradovic Z Uversky VN The unfoldomics decade: an update on intrinsically disordered proteins BMC Genomics 2008 9 Suppl 2 S1 18831774 10.1186/1471-2164-9-S2-S1 PMC2559873

